# Double Inguinal Strangulated Hernia in a Cryptorchid1Read before the Medical Society of the County of Erie, February 20, 1911.

**Published:** 1911-04

**Authors:** L. G. Hanley

**Affiliations:** 428 Porter Avenue


### Double Inguinal Strangulated Hernia in a Cryptorchid.[1]

BY L. G. HANLEY, M D.

THE dangers and worry caused by malformations or defects of the genitalia produce an annoying condition from psychic and physical viewpoints.

Among genital abnormalities are misplaced or undescended testicles which if not treated surgically frequently produce in the patient a constant state of uneasiness. It is estimated that undescended testicles exist on an average of one in every five hundred. The discomforts and dangers attending a monorchid or cryptorchid include hernia, severe twisting of the cord, atrophy, epididymitis and probability of injury and subsequent malignant disease.

June 20, 1910, I was called by Dr. Pierce Candee to see Mr. J. who had been suffering from strangulated hernia for twelve hours. Frequent unsuccessful attempts at reduction had been made prior to my arrival. When first seen he was vomiting stercoraceous material and was in great agony throwing himself from the cot to the floor and crying out loudly. Hernia had been down several times before on the right side he informed me, but had always been easy of reduction. I advised him to go to the hospital immediately and be prepared for operation if under an anesthetic reduction were impossible. This he refused to do unless all other means failed. Chloroform was administered and with difficulty he was placed under its influence. I tried for about ten minutes to reduce the bowel by taxis and had asked his father to send for the ambulance when I felt the mass begin to recede and enter the abdomen. This was on the right side. The hernial mass extended upwards to the anterior superior spinous process and measured seven by four inches. The hernia on the left side was about four by four inches. This was easily reduced. On either side was a testicle outside of the external ring. The one on the right could be moved under the skin and superficial fascia of the external oblique for a distance of about two inches. Scrotum empty. Testicle on left side was at the opening of the external ring and only slightly movable. He went to the Sisters' Hospital the next day and was prepared for the operation, which was done June 25, 1910. There was considerable ecchymosis of the tissues over the site of swelling and lower one-third of the penis caused by the strangulation and taxis. The superficial

---

1. Read before the Medical Society of the County of Erie, February 20, 1911.

fascia and aponeurosis of the external oblique were cut when the testicle could be seen. It could be moved freely and be pushed back through the ring for a short distance into the abdominal cavity. The peritoneum did not extend far into the scrotum and it was necessary to cut the cremasteric muscular fibers and the transversalis fascia and free the cord and adhesions. The vaginal portion of peritoneum above the testicle was cut transversely, and the upper end closed with iodized gut sutures. A purse string suture closed the portion of peritoneum near the testicle. By wiping the peritoneum with a piece of gauze I was able to break up all retarding fibers. A cavity was then made in the scrotum with blunt scissors and my fingers separating the structures by stretching sufficiently large for the testicle into which it was sutured. Beginning at the bottom the lower part of the testicle was fixed with iodized gut to the scrotum, stitches penetrating the testicle. A herniotomy was then done leaving the cord in place. Recovery was uneventful.

November 4, 1910, the patient returned to the hospital and an operation was performed on the left side. There was not so much play for the cord as on the right and I was obliged to cut the spermatic vessels to get sufficient descent for the testicle; otherwise the technic was the same as on the right side. Both testicles are now in the scrotum and both hernias cured.

I saw him one month ago and with the exception that the right testicle is a trifle lower than the left he is perfectly normal. An ordinary hernia is a great source of danger, and when accompanied by an undescended testicle is still greater. This case seems to be unique as there was a double hernia, strangulated in a cryptorchid. If the testicle can be brought down without tension a good result is assured.

428 Porter Avenue.

